# Report from the Medical Library Association’s InSight Initiative Summit 2: Meeting the Evolving Information Needs of Library Stakeholders

**DOI:** 10.5195/jmla.2019.669

**Published:** 2019-04-01

**Authors:** Katherine G. Akers

**Affiliations:** Editor-in-Chief, *Journal of the Medical Library Association*, and Biomedical Research and Data Specialist, Shiffman Medical Library, Wayne State University, Detroit, MI, katherine.akers@wayne.edu

## Abstract

At the Medical Library Association’s InSight Initiative Summit 2, held September 27–28, 2018, academic and hospital librarians joined with publishing industry partners to develop a deeper shared understanding of technology- and social interaction–driven changes in how health sciences researchers and clinicians discover and consume information in their fields. Through a mixture of keynote talks, a panel discussion with health care professionals, and small-group problem-solving exercises, the summit program invited participants to collaboratively develop strategies for helping users recognize the value of curated or peer-reviewed content obtained through institutional access channels. Themes of the summit included the existence of different user modes of information discovery and access, user reliance on professional societies and Twitter as information sources, the extent to which smartphones are used to find medical information, the importance of inducing disorienting dilemmas in library users that cause them to recognize librarians as true partners in information seeking and research, the dangers of depending on non-curated information, and the need for publishers and librarians to work together to ease barriers to access and enrich the user experience.

The Medical Library Association (MLA) InSight Initiative Summit 2, held September 27–28, 2018, in Chicago, Illinois, brought together library leaders and publishing industry partners to engage in high-level, high-value dialogue on issues of common interest that impact the health information profession. The theme of this summit, “Meeting the Evolving Information Needs of Library Stakeholders,” addressed technology- and social interaction–driven changes in how medical students, residents, physicians, nurses, and researchers discover, access, and evaluate health sciences information to identify fertile ground for collaboration between medical librarians and publishing industry partners. The program included a mixture of keynote talks, a themed panel discussion, and small-group problem-solving exercises.

## WELCOME AND SMALL GROUP EXERCISE #1

Dan Doody, summit facilitator, and Michelle Kraft, AHIP, liaison to the MLA InSight Initiative Task Force, welcomed InSight Summit 2 participants and explained that, in addition to learning from and engaging in summit activities, an important subtext of the summit was networking with colleagues, with particular value placed on connections formed between librarians and representatives of publishing industries. They explained how a key outcome of Summit 1, “Engaging Users in a Disruptive Era” [1], was the realization that for health sciences libraries and publishing industries to maintain critical roles in the publication and dissemination of information into the future, their common focus must be on the user. Thus, the theme of Summit 2 was crafted to allow a chance to dig more deeply into understanding health sciences users’ information-seeking needs and habits.

Doody and Kraft thanked the Summit 2 program committee members for their work and the Association of Academic Health Sciences Libraries (AAHSL) and Elsevier for financial contributions. The welcome address was followed by an ice-breaker exercise allowing summit participants to learn more about each other.

## KEYNOTE TALKS

### Keynote #1: Understanding the user, a quantitative perspective: what users tell us about their information discovery and consumption habits

#### Tracy Gardner, Principal Consultant, Renew Publishing Consultants

Renew Publishing Consultants has conducted online surveys of the information habits of scholarly researchers since 2005. Starting as a small survey of how life science researchers in the United States access journal articles, it has since expanded to encompass researchers from all disciplines from around the world and to consider a wider range of information sources, including books and academic videos. Renew Publishing Consultants’ most recent report, “How Readers Discover Content in Scholarly Publications” [2], is freely available online along with supporting data to allow further analyses. The 2018 survey was sponsored by several publishing organizations, including the JAMA Network, PLOS, Sage, Wiley, and TrendMD.

Gardner focused her talk on survey responses from medical professionals (i.e., practitioners involved in patient care). She found that when looking for journals articles, North American medical professionals relied less on abstracting and indexing (A&I) databases (e.g., PubMed, Web of Science), academic search engines (e.g., Google Scholar), and social or professional networking sites (e.g., ResearchGate, LinkedIn) and more on professional society web pages, compared with academic researchers. European medical professionals used A&I databases more and society web pages less than their North American counterparts, prompting the question: Why do North American medical researchers not have a strong preference for A&I databases?

When asked how they discovered their most recently read journal article, only 33% of medical professionals reported a literature search, compared with 40% of academic researchers. Rather, medical professionals were more likely to discover articles through recommendations that they received through email, prompting the question: Who is smarter? Users who perform their own searches or those who socialize their article discovery by relying on others’ recommendations?

Compared with librarians, researchers across disciplines were less likely to start their searches for a journal article through a library web page or journal aggregator and more likely to use an academic search engine (e.g., Google Scholar). Furthermore, master’s degree students were most likely to start their searches for a journal article using a general search engine (e.g., Google), prompting the questions: Are researchers just rebels? Are master’s students uneducated in literature searching?

When asked what proportion of journal articles were accessed from difference sources, North American medical professionals relied most on publisher or journal websites or full-text aggregators; European academic researchers relied most on free subject repositories (e.g., Europe PubMed Central); and Chinese researchers relied most on SciHub, prompting the question: Is the North American academic sector better funded or are their authentication methods (e.g., Internet protocol [IP] authentication) less of a barrier? Furthermore, researchers reported little use of tablets or smartphones to access journal articles, regardless of their sector or geography, prompting the question: Why are we so worried about mobile delivery when it is barely used for reading journal articles?

Based on the survey results, Gardner described five different “pathways” through which researchers access journal articles.

1. *Traditional:* Users begin at a library website, navigate to an A&I database, search the database, and click through to articles.
2. *Researcher-focused:* Users follow the interests of other researchers (e.g., through social media or reference lists in published articles).
3. *Random:* Users simultaneously search in multiple databases until they find relevant articles.
4. *Cover all the bases:* Users perform logical, thorough searches that employ multiple information sources in a sequential manner (e.g., academic search engines, review of reference lists in published articles, colleague recommendations).
5. *Creature of habit:* Users always rely on their preferred information sources (e.g., ResearchGate, Google Scholar).

When asked which features of publisher or journal websites researchers found most useful, academic researchers reported liking author services (e.g., online manuscript submission system, information for authors), whereas medical professionals reported liking links to related articles.

To find academic videos, academic researchers were more likely to use YouTube, whereas medical professionals were more likely to use society web pages. Regarding their most recently watched video, academic researchers were more likely to have searched for the video, whereas medical professionals were more likely to have found a video associated with a journal article. There was little difference between medical and academic sectors in how people accessed books, although those in the medical sector were again more likely to go to a society web page.

Together, these survey findings led Gardner to wonder what people in the medical and academic sectors could learn from each other and, in particular, how professional society relationships are involved in the search for and discovery of information.

### Question-and-answer session

During the question-and-answer session, a librarian asked which personalization features on publisher or journal web pages were least valued, regardless of medical or academic sector. Gardner answered that the least valued features were those that required users to create an account on the publisher or journal website, such setting up email alerts for recently published articles or seeing a history of articles they have read. Garner speculated that navigating different personalization features on different websites is cumbersome and might be confusing to users with institutional access.

Audience members who were representatives of professional societies were asked to remark on medical professionals’ reliance on society web pages. Society representatives expressed varying levels of agreement about whether their members tended to use their web pages as a first place to find information. One person supposed that, as many societies publish their own journals, users might view society websites and publisher or journal websites as being one and the same. Supporting this possibility, Gardner said that she observed a correlation between “publisher/journal website” and “society website” responses from survey participants. Furthermore, she speculated that the societies supporting the Renew Publishing Consultants’ survey might have influenced who participated in the study, possibly increasing the number of participants with established connections to a society.

When asked about the biggest surprise in the 2018 survey results, Gardner said they expected A&I databases to be less important than in the past, but they did not see much evidence of this overall. Rather, they found that A&I databases were still highly used by researchers, indicating their relevance.

Audience members also posed several questions sparked by the survey results, such as whether information-seeking behavior differed by age or career stage, whether the more advanced systematic reviewing in Europe might explain differences in information-seeking behavior between European and North American researchers, and whether medical professionals relied less on A&I databases because they were more likely to seek summarized answers to clinical questions.

### Keynote #2: Understanding the user, a qualitative perspective: how librarians understand and adapt to the evolving needs of users

#### Jeff D. Williams, AHIP, Chair of the Department of the Medical Library and Director of the Health Sciences Library, New York University (NYU), NYU Lagone Health

To understand how librarians at the New York University (NYU) Health Sciences Library have adapted their services to respond to users’ changing needs, Williams asked his librarians several questions in a focus group setting, including: What are some changes in the needs and behaviors of library users over your career? How have you adapted your approach as an information professional, based on these changes? Can you describe any experiences that caused you to reexamine fundamental assumptions about users’ needs and behaviors? What are misconceptions that create barriers between the library and its users?

By performing textual analysis of focus group transcripts, Williams identified five major themes related to evolving user needs.

1. *User behaviors:* Whereas obtaining scholarly information used to be a discrete activity (e.g., a visit to the library), it is now integrated into users’ daily workflow. Today’s users have more experience and comfort with technology but are less patient with or tolerant of complex systems for access.
2. *User needs:* Some users want assistance with data management, research, and publication metrics, and they want their immediate needs met as quickly and easily as possible due to time pressure.
3. *Spaces:* Users continue to value physical library space, which remains intertwined with library services, although they do not need to enter that space to access information.
4. *Information resources:* Information resources are seen as “academic infrastructure” (or “academic electricity”) that is not grounded in the physical space of the university.
5. *User misconceptions:* Users think that complex information questions can be answered over chat or email, see librarians’ role as merely providing clerical or administrative support (e.g., delivering portable document format files [PDFs]), and fail to see the difference between vendors and publishers.

In his analysis of focus group data, Williams detected several paradoxes in users’ information-seeking needs and their perceptions of the library. For example, some users ask for instruction in certain areas, whereas others are frustrated by the need for such instruction. Also, some users enthusiastically suggest additions to library services, but others struggle to understand new, nontraditional library services (e.g., data management support).

Williams has also noticed paradoxes between library user needs and perceptions during the strategic planning processes at the NYU Health Sciences Library and University of California, San Diego Library. At both institutions, users often suggested that the library develop services that were already offered and were surprised that the library provided services that librarians assumed everyone would know about. Although this could indicate a problem with library marketing, Williams suggested that it reflected something different: a cognitive barrier in users’ views of libraries. He found that many library “power users” assumed that the library was a “big room with books” before gaining a full recognition of the breadth and depth of library services. Williams asked, “What got the user past this cognitive barrier? Not marketing. Not an outreach event. Not a presentation at a faculty meeting. The user had a disorienting dilemma.”

The term “disorienting dilemma,” from Jack Mezirow’s transformative learning theory, refers to the state in which a person experiences something that does not fit their implicit expectations or individual worldview. Williams observed that the catalyst often causing a user’s disorienting dilemma—and resulting transformation in their perspective of the library—was working closely with a librarian in the user’s own work environment. Such a user might say, “I had no idea librarians knew about [research methods, systematic reviews, or structuring research data], but now I work with a librarian all the time.” Williams believes the key impetus for inducing disorienting dilemmas in library users is the act of librarians getting out of the library and becoming part of their user communities. In conclusion, Williams described the MLA InSight Initiative as another type of disorienting dilemma that could break down cognitive barriers between librarians (“us”) and publishers (“them”).

### Question-and-answer session

A librarian asked Williams to comment on the impact of Superstorm Sandy, which destroyed the NYU Health Sciences Library in 2012. Williams answered that because librarians did not have a physical library as an anchor, they had to come up with different ways of working and “lean in to discomfort,” which led to the formation of meaningful connections with library users. Another librarian described disorienting dilemmas as a “chicken and egg” situation: if librarians cannot get their “foot in the door,” how can they induce a disorienting dilemma? Williams explained that when librarians think about what activities they should prioritize, they should focus on activities that engage them with users. Getting power users to recommend the library to their colleagues would also be useful. A publisher asked why there were not more embedded librarians if embeddedness was the key to developing and maintaining relationships with users. In response, librarians described some challenges of physical embeddedness, such as the existence of different cultures in different areas of research or practice, a potential loss of professional connections with librarian colleagues, and library staffing issues.

A few librarians spoke about the change from librarians being service providers to being partners in research and information seeking. One remarked that saying, “I can do research for you,” failed to connect with nurses, whereas saying, “I can help you find information,” was more effective in getting nurses to turn to librarians. A hospital librarian said that it is important for librarians to anticipate future changes that could influence health care decision makers and to be prepared to say, “We can help you with that” or “We can help you do your job more easily.” Another librarian, reflecting on disorienting dilemmas, stated that “when you find a paradox, you are getting close to the truth” and described today’s librarians as no longer merely occupying a support position but instead becoming partners or collaborators with researchers and clinicians. Williams agreed that “a paradox is a springboard to looking more broadly at a topic.”

Some publishers asked how they could break down barriers between themselves and librarians or library users. Regarding connecting with librarians, Williams said publishers have to work together with librarians to solve common problems and find vehicles for bringing librarians (and library users) in-house to provide perspective on their products rather than just money. Regarding connecting with library users, Williams suggested that publishers could visit campuses to give product demonstrations to library users. Finally, a publisher speculated that the vast number of different publishers was a problem and that a collaborative organization might be better for users. Williams agreed that a collaborative approach would improve efficiency around specific issues, such as access to licensed content.

## SMALL GROUP EXERCISE #2

Participants gathered in small groups with roughly equal representation from librarians and publishing industry representatives to discuss how librarians and information providers could collaborate to (1) support users through the research and publishing life cycle and (2) improve users’ information literacy. The groups defined broad problems, identified areas of concern or controversy, and suggested next steps for moving forward.

### Suggested conversation starters

#### Researchers or authors and the library

- An InSight Initiative Summit 1 keynote talk suggested that for librarians to remain relevant, they must insert themselves in all stages of the research and publishing life cycle. What are those stages? How do we define “author?” How can librarians realistically insert themselves as a partner at each stage?
- Can librarians assist in citation management, maintenance of individual scholarly repositories, or similar functions? Should they try to do so? If so, should they enlist information providers’ help in this effort?
- Can or should librarians and information providers be involved in facilitating researchers’ presence on social media (e.g., Twitter, ResearchGate)?
- Are there other ways that librarians can be true partners with researchers and authors of professional content?
- What kind of publishing-related educational programs can information providers offer to libraries to sponsor for their users (i.e., current and future authors)?

#### Users and information literacy

- What are the milestone “cradle-to-grave” steps in the careers of health care professionals? What can publishers and librarians do to support users in each of these steps?
- How do or should information providers and librarians improve the health information literacy of new students through established researchers?
- Which resources are most valuable to librarians in providing help? Are there resources that fall short in terms of what users need?
- How can librarians utilize or collaborate with information providers to help health care professionals discover and access information and information tools for desired outcomes? Is there a need for additional tools, and what would their functions be?

#### Group 1

It is important to understand the career stage of researchers to determine what type of help they need. For example, as researchers become more senior, they may need guidance in being a peer reviewer, which could incorporate evaluative feedback from authors on review quality. Researchers could also benefit from industry-standard information about author fraud (e.g., deliberate altering of scientific images). Relationships between librarians and publishers could be improved by being more careful in how we talk about each other (e.g., referring to publishers as “evil”), because negative sentiments can trickle down to users. Publishers could reach out to librarians, who often no longer have purchasing power, to provide or create useful tools and services. For example, publishers could collaborate with librarians in creating brief topical videos for users at the point of need.

#### Group 2

Approaching the bench researcher community is more challenging than approaching the clinical practitioner community. Publishers could help by providing focused training around specific resources. For example, vendors could create “bite-sized” instructional materials (e.g., forty-five-second videos) for users, visit campuses to train new librarians in using their resources, provide practical data on usage at particular institutions (instead of performing “road shows”), and attend library-hosted technology fairs. Publisher-librarian partnerships could help librarians break into other stages of the research process, instead of only end stages. Publishers and librarians should find common ground, such as working together to dispel myths about open access, while being cautious about user assumptions, as even doctoral (PhD) researchers do not know everything.

#### Group 3

As the socialization of information literacy occurs across a lifetime—from high school students to undergraduate students to graduate students to professors—we must interject at each point to change the path. As everyone has a different perspective of the research life cycle, we must talk to each other to determine where our life cycles overlap. Publishers and librarians could partner in designing new resources, such as those related to research rigor and reproducibility. For example, publishers and librarians could collect and share “redemption stories” of research projects that went wrong but were fixed.

#### Group 4

The research and publishing life cycle is extremely long, with many opportunities for insertion of librarian-publisher collaborations. Librarians already play many roles in publishing, including being authors on systematic reviews, helping authors identify journals in which to publish, editing manuscripts, and creating bibliographies. Publishers could promote these librarian roles on their journals’ web pages that provide information for authors. In turn, librarians could be more aware of and refer authors to publisher resources such as Cambridge University Press’s Author Hub, which helps researchers and clinicians become better writers through in-person workshops and free editing services for authors from low-income countries. Better user interfaces for journal websites could lessen user reliance on article pirating sites such as SciHub. However, the rapid pace of science spurs questions over the relevance of the current publishing model and whether preprint servers might be better suited for scientific communication.

#### Group 5

As the licensing of images is a problem area in publishing, librarians and publishers could work together to overcome barriers due to copyright issues by streamlining the licensing of content. Publishers could also form stronger relationships with librarians by providing resources that are helpful to both librarians and users (e.g., short videos, learning tools) and by customizing emails that are sent to individual librarians at particular institutions. The “elephant in the room,” however, is Google. To remain relevant, it is important for librarians to leverage the growing relationships between publishers and Google.

#### Group 6

Librarians can provide many services that support publishing, including assisting with writing, hosting writing centers, helping authors choose journals that are topically appropriate and reputable, depositing journal articles and data in repositories to meet funder requirements, and promoting recently published journal articles through social media. Publishers can support authors by providing writing workshops, being present at scholarly conferences, and providing training on how to be a peer reviewer and critically appraise journal articles. Potential areas for librarian-publisher partnerships include writing workshops and citation management.

#### Group 7

Relationship-building is critical. It is necessary to tease out different modes of access such as “browse mode” or “search mode”—even within more narrow categories, such as students or faculty—and help users in each mode. Librarians need to talk directly with users to determine actual needs, instead of making assumptions. Publishers could help by more proactively contacting librarians about their usage statistics, incentivizing users to come in and learn more about the library (e.g., publisher sponsorship of events), supporting the work done by librarians to educate users, and incorporating author ORCID identifiers into their workflows.

## DINNER

During dinner, medical residents—most of whom practiced family medicine—sat at each table to allow librarians and industry representatives the opportunity to talk directly with these young health care providers about their information-seeking practices. After dinner, each resident briefly described the highlights of their conversations, which included distinguishing between evidence-based medicine and “experience-based” medicine, learning about different sources of and approaches to accessing medical information, recognizing differences in the perspectives of librarians and publishers, and realizing that librarians can round with clinicians to provide immediate answers to clinical questions. The residents expressed appreciation for the chance to connect with librarians and thanked the librarians for their work.

## PANEL DISCUSSION

### How users really discover and use library resources: how do they learn; how do they keep up to date; how do they interact with peers to foster learning; what devices do they use; what works about the present system; what does not work?

The panel discussion featured personal experiences related by five health care professionals on how, where, and on what devices they discover, access, and consume information in their fields. The panelists represented a variety of types of health care professionals at different career stages so that summit participants could receive varying testimonies on the changing information needs of a cross-section of users.

#### Resident: Allison Lale

Allison Lale recently completed two residencies in radiology and family medicine, which she described as very different practices with different types of sources of and approaches to consuming information. During her family medicine residency, Lale stayed up to date with literature in her area by participating in weekly didactic sessions (i.e., monthly journal club with *Evidence-Based Practice* articles) and precepting medical students. While in the clinic, she preferred using her smartphone over a computer to look up answers to “on-the-spot” clinical questions in UpToDate, Epocrates, LactMed, the Electronic Preventive Services Selector (ePSS), the Centers for Disease Control and Prevention’s Contraception app, and PediCalc and MDCalc apps. She took screenshots of websites and apps and pictures of journal articles or presentations to save for later reference. When she needed to perform in-depth research, Lale used UpToDate, various apps, and online question banks; printed out articles from journals (e.g., *American Family Physician*); and reached for textbooks and board exam prep books (e.g., *Essentials of Muscduloskeletal Care*, *Harrison’s Principles of Internal Medicine*). Lale described using her medical library to access electronic journal subscriptions and asking librarians for help with literature searches.

#### Nursing administrator: Janice M. Phillips, Rush University Medical Center

Janice M. Phillips described her primary job as helping nurses perform research and evidence-based practice. Her favorite journals were *Nursing Outlook* and *Health Affairs,* and she was an avid reader of newspapers and user of National Library of Medicine resources. Despite having access to the literature through her current university, Phillips also paid an alumni fee to continue accessing content through her previous institution. Phillips attested to being a lover of libraries and librarians. She described a strong partnership between her unit and the nursing librarian, who attended monthly nursing research meetings to offer resources and services, created a custom “Library Orientation for Nursing LibGuide” containing links to evidence-based practice resources and books, and hosted an online journal club for nurses. Phillips also visited her public library on a weekly basis.

#### Allied health clinician and researcher: Margaret Danilovich, Feinberg School of Medicine, Northwestern University

Margaret Danilovich is primarily a physical therapy researcher, but also engages in administrative tasks and teaching. Her main method of keeping up with the literature and primary point of connection with other researchers was Twitter, on which she spent five to ten minutes of every hour. She used Twitter to follow specific funding agencies and journals, which allowed her to learn about new funding opportunities and calls for submission to special journal issues. She also discovered recently published journal articles using BrowZine and Google Alerts and browsing abstracts in print journals she received through society memberships. Danilovich has received research support from medical librarians who have performed literature searches and imported the retrieved records in Covidence to help her “plow through” abstracts in preparation for grant applications, resulting in high scores on her literature review component from grant review committees. In her teaching duties, she has worked with librarians to teach EndNote and PubMed searching, obtain full-text articles for posting in course management systems, use UpToDate in place of textbooks, and prepare book-free curricula for doctoral students. She said that librarians at her institution also taught helpful courses on using R software for statistical analysis, EndNote, best practices in research data management, and biosketch preparation.

#### Internal medicine physician: Vineet Arora, Pritzker School of Medicine, University of Chicago

Like the previous panelist, Vineet Arora described using Twitter on her smartphone as her primary method of keeping up with the literature. As the social media editor for the *Journal of Hospital Medicine*, Arora hosted a monthly hour-long Twitter-based journal club (#JHMChat) to connect clinicians, educators, researchers, and patients around particular topics by focusing on a recently published article. In an analysis of #JHMChat participation, she found that each chat session resulted in two million impressions (i.e., number of times content is displayed to users) and led to a spike in journal article page views [3]. When she worked in a clinical setting, Arora found information using UpToDate on her smartphone and literature searches in PubMed. In reflecting on how she taught residents to keep up with the literature, Arora acknowledged that time pressures made it difficult to find answers to clinical questions arising in daily practice. She described her study showing that embedding a clinical librarian in rounds increased the number of clinical questions asked and answered, the amount of time spent discussing questions, and question quality (i.e., patient intervention comparison outcome [PICO] format) without affecting the time spent rounding [4]. Residents who rounded with a clinical librarian reported that it gave them more confidence in asking and findings answers to clinical questions and led to changes in patient care.

#### Pediatrics researcher and clinician: Michael Msall, Pritzker School of Medicine, University of Chicago

Michael Msall is a professor of pediatrics who spoke about the need to bypass misinformation and integrate reputable biomedical, educational, and social information to improve outcomes for vulnerable children. He discussed common “myths” of premature births, including the belief that children born prematurely will be permanently disabled—incapable of walking, seeing, hearing, communicating basic needs, or learning in peer groups—in part due to stories such as *Life* magazine’s “Born Too Soon” [5]. In fact, randomized controlled trials demonstrate the efficacy of interventions for premature infants (e.g., maternal steroids, lung surfactants). Today, the vast majority (80%) of prematurely born children do not exhibit neurodevelopmental disabilities; at age 5, 97% can walk and talk, are potty-trained, and can dress themselves, and only 2% are dependent on medical devices or drugs. Another myth is that parental age and education are the primary drivers of premature birth, whereas the major contributor is poverty. Msall bemoaned trust in Google and famous people as sources of health information and lauded the book *Bad Advice: Or Why Celebrities, Politicians, and Activists Aren’t Your Best Source of Health Information* [6] by Paul Offit, an infectious disease researcher and advocate for childhood vaccinations.

### Question-and-answer session

Summit participants asked the panelists which aspects of Twitter worked really well for them and how they transitioned to using Twitter as their chief discovery tool. One panelist said that Twitter has wide reach: “if you publish an article and don’t talk about it, no one reads it.” She said that she has always used social media to stay abreast of information in her field and that using Twitter expanded her perspective. Another panelist said that more people are entering medical professions with the expectation that social media is the norm for information dissemination. She said that her residency programs had social media accounts for resident recruitment, education, and recognition and that they embedded Twitter feeds on institutional web pages. Another panelist said that she did not grow up with social media but found that after “diving into Twitter” and establishing a social network, “Twitter does the work for you.” Because a single person cannot keep pace with the rate of scholarly publishing in any given field, Twitter acts as an “information curation tool” that relies on a “network of people you trust and respect.”

A publisher asked a panelist how much a journal title mattered when they decided what to read. One panelist said that they did rely on a “hierarchy of [journal] reputation.” Another panelist agreed that “prestige matters” and stated that higher impact journal made more effort to publicize their content, but smaller journals could use social media to actively engage their readers and authors. Another panelist said that she gravitated toward interdisciplinary journals and found really good articles in smaller journals, stating that “I have to cast a broad net to get what I am interested in.”

A librarian asked about how panelists’ colleagues who were not affiliated with academic institutions gained access to journal articles. One panelist said that there was a “black market” for journal articles and that colleagues sent PDFs to each other. Another panelist suggested that alumni of an institution should be able to maintain access to core resources (i.e., “access lite”), and other panelists agreed that alumni could be stronger advocates of continued access and could make donations to their graduating institutions to retain access.

The conversation turned to how to facilitate the discovery of information by health care practitioners. A librarian asked how panelists balanced the immediacy of information access with the lifelong, deeper learning skills that were necessary for improving medical practice. A panelist stated that she did not know whether the medical information taught today “will hold the test of time” but that lifelong learning skills, such as the ability to critically appraise literature, would persist throughout one’s career. She also said there was a role for the smarter use of electronic health records, which could build in learning and reporting tools to change clinical practice. Another panelist said that physicians did not have time to evaluate the primary literature but instead relied on updates from professional societies with the assumption that those societies appropriately vetted information. A participant lamented that librarians often tried to help with information overload by giving users even more information and asked, “How can we hand you the most pertinent, current information for your day?” The panelists replied that they would like to receive information in the form of annotated bibliographies in newsletters, one-sentence summaries of journal articles (e.g., *New England Journal of Medicine [NEJM]* Journal Watch), podcasts, games (e.g., question of the day, *NEJM* Image Challenge), bulletins containing practical guidelines (e.g., “do this, stop doing that”) with supporting references, or very brief and direct videos.

## SMALL GROUP EXERCISE #3

Participants gathered in small groups with roughly equal representation from librarians and industry partners to discuss how librarians, publishers, and providers of discovery and management tools could collaborate to more effectively communicate the value of information. Groups developed persuasive statements to explain the superior value of the information that they collaborated on to provide the user “free” information, as well as how counterproductive it was to use or rely on “pirate” sites and predatory publishers.

### Suggested conversation starters

- What arguments can be made to explain to users why curated resources (e.g., journals, book, A&I databases) are more valuable than “free” (e.g., advertising-supported, social media) content?
- Given conveniences such as single sign-on and proxy servers, are users generally aware when they are using resources that are curated and made available by the library? Are there ways to make the library’s involvement clear without inconveniencing users? What more can publishers do to help libraries make patrons aware of the fact that this is valuable content paid for by the library? What can information providers do to help libraries communicate the value of these paid resources? What more can libraries do to publicly recognize the role of publishers and library or content service providers?
- Users sometimes say they receive content “free from the library.” Without jeopardizing nondisclosure agreements, how can libraries communicate the high cost of providing many types of content?
- The overall goal is to get users to recognize—and avoid—outlets labeled as “pirate” sites and predatory publishers. How do we collaborate in making sure users recognize this is an important goal for them (and not just for us)?

#### Group 1

It is unclear whether users do not understand the value of curated information or the fact that libraries pay to access that information. We must break down barriers to accessing licensed content and make better use of Google subscriber links. We could change the language used on our resources (e.g., “brought to you by…”). “Google” is used as a verb; could “library” also be a verb? Publishers and librarians could work together to teach authors about predatory publishing, including distinguishing between predatory versus new journals and understanding the damage that can result from publishing in or using information from predatory journals.

#### Group 2

We must identify and raise awareness among all stakeholders of the dangers of publishing in predatory journals and using non-curated content. Librarians must maintain engagement with users, and publishers could help by developing case studies about the pitfalls of publishing in predatory journals, without resorting to fear-mongering. It is important to get early career researchers on board, such as by tapping into established researchers for their testimonials.

#### Group 3

The role of librarians is to help users recognize which resources they should use and develop critical appraisal skills. This role can be enhanced by partnering with publishers. We should recognize that researchers use Twitter to follow the literature in their field and that clinicians use apps that their peers recommend and rely on notifications of advances by specialists in their field through professional societies. As society recommendations do not always align with recommendations found in point-of-care tools, it would be good for these tools to provide disclosures.

#### Group 4

Premed and early medical students tend to use the easiest methods to find information (e.g., Google). We must “meet them where they are” and focus on essential appraisal skills. Because Google does some things really well, we should learn from them. For example, we need to make it easier to access subscribed content, as it can take up to five minutes to retrieve an article through institutional access routes. We should also teach students about the benefits of peer review and use of information from trusted sources. Librarians could publish commentaries in nonlibrary journals about new resources, tools, and skills, such as case studies of what happens when people use bad resources. We have “a lot of carrot power but not a lot of stick power.” We must “clue people in” that librarians are here to help, which could involve institutional branding of resources to remind users that they are using the library or informing users about the cost of library resources.

#### Group 5

Can we talk to Google about moving reputable sources up in search results, such as by filtering out predatory journals? How do we effectively teach critical appraisal skills to users? Librarians could work with publishers to raise awareness of smaller specialty journals and create fun content, such as games and data visualizations, for readers. Publishers could help by developing training tools and web page plug-ins, creating library branding, and alerting librarians about retracted articles.

#### Group 6

Users do not always know what libraries pay for or the differences between crowd-sourced versus peer-reviewed resources or quick look-up versus curated information. Smaller journals are still important even though they have lower impact, and publishers often find the 5-year impact factor to be most useful. Authors need funds to pay for open access publishing, and the embargo period should be shorter for hybrid journals. Some unanswered questions are: Will smaller presses survive in an open access world? Do we need iTunes pricing for journal articles? Participants were encouraged to read the *Scholarly Kitchen* blog post “Focusing on Value—102 Things Journal Publishers Do” to prove they add value beyond peer review, copyediting, and formatting [7].

#### Group 7

We suspect that users do not care about where they get information; rather, “they want what they want when they want it” and will not use content that they cannot access. As some research cultures do not view librarians as professionals, librarians sometimes experience difficulty in being viewed as research partners. Rather, researchers are more trusting of professional societies and their peers and may use SciHub instead of accessing content through libraries or publishers. Users would benefit from the standardization of platforms that host licensed content across systems. A paradox emerges, however, when we want to not only make access seamless and invisible to users, but also want them to know that the library pays for that access. We should start talking about publishing in journals that are right for the author as opposed to focusing on predatory journals.

## PLANNING SUMMIT OUTCOMES

Facilitated by Kraft, this session drew all participants together to collectively identify critical ideas that could stimulate the development of enduring materials that advance the cause of user engagement and of medical libraries in general. The goal was for some participants to leave the summit with a mandate to spearhead the creation of specific materials for wider dissemination.

Kraft thanked the summit planning committee for choosing the articles in the pre-summit reading list and the small-group scribes and summit facilitators for fostering conversations among participants. She noted that an official summit report would be published in the *Journal of the Medical Library Association*, and she obtained two volunteers—one librarian and one publisher—to write a *Scholarly Kitchen* blog post [8]. She also explained MLA’s intention to hold an eighty-five-minute immersion session at MLA ‘19, focusing on a single summit theme.

Kraft asked participants to share their desired summit outcomes and ideas for communicating lessons learned to others in the form of enduring intellectual property. One librarian said, “we need to talk to Google” and invite a Google representative to Summit 3 to talk about their algorithms. Other librarians suggested that librarians and publishers could cowrite a series of case studies about research going awry but being resolved or could repackage direct user testimonies about information seeking at a conference.

Doody suggested that user testimonies could be the topic of an MLA ‘19 immersion session and that other ideas for thoughtful immersion sessions could be how to make MLA vendor exhibits more engaging and educational experiences for librarians or a report of themes from the most recent Renew survey, with accompanying librarian responses. A publisher asked how they could take these discussions back into their industries. Another publisher said, “I find that we need each other. We shouldn’t be competing with each other. There is not a winner and a loser. We can better educate our businesses on your librarian world” and help show users the value of the content that libraries license. Doody and Kraft suggested that a publisher-oriented infographic or “take-away package” might be helpful toward this end.

## SUMMARY AND FUTURE STEPS

Doody reiterated some key questions raised in the summit. Based on the data that Gardner shared from the Renew survey, are publishers and librarians spending too much time and attention on pirate sites like ResearchGate and SciHub and on delivery of content through mobile devices? Based on what participants learned from the panelists, some users are highly dependent on their smartphones for accessing information. What are the take-aways from this? What are the implications of traditional A&I databases maintaining a leading role in information discovery? As librarians, how can we induce disorienting dilemmas in our users? As publishers, how can we induce disorienting dilemmas in our authors and readers? How can we systematically use library evangelists to help communicate our value proposition? Librarians say that engaging users is easier in some areas (e.g., nursing) and more difficult in other areas (e.g., basic sciences). What are some concrete ways that information providers can help librarians with user outreach? What are the implications of health sciences researchers and clinicians using Twitter and other social media tools for information dissemination and discovery? Finally, how can information providers and librarians collaborate to more effectively communicate the value of the information that they produce and provide?

Doody thanked the publishing industry sponsors for their financial support of the summit and said that their continuing support would be requested in 2019. He solicited three librarians and three industry partners to serve on the program committee for InSight Initiative Summit 3. He invited all participants to a reception for MLA leaders and an open forum discussing Summit 2 at MLA ‘19. He stated that Summit 4 is also being planned and thanked Kevin Baliozian, MLA executive director, and Mary Langman, MLA staff liaison, for their contributions to the MLA InSight Initiative.

## CLOSING

Baliozian thanked Doody and Rich Lampert, summit co-organizers, for their work in planning and facilitating the summit and being “true believers” in the MLA InSight Initiative. He wished participants safe travels back home and hoped to see many again at MLA ‘19 or the next summit.

## PRE-SUMMIT PARTICIPANT READING LIST

Booth A. In search of the mythical ‘typical library user.’ Health Inf Libr J. 2018 Sep;25(3):233–6. DOI: http://dx.doi.org/10.1111/j.1471-1842.2008.00780.x.
Boruff JT, Storie D. Mobile devices in medicine: a survey of how medical students, residents, and faculty use smartphones and other mobile devices to find information. J Med Libr Assoc. 2014 Jan;102(1):22–30. DOI: http://dx.doi.org/10.3163/1536-5050.102.1.006.
Carpenter TA, Luther J. Failure to deliver: reaching users in an increasingly mobile world. Scholarly Kitchen [Internet]. 15 Jun 2017 [cited 18 Sep 2018]. <https://scholarlykitchen.sspnet.org/2017/06/15/failure-to-deliver/>.
Chong HT, Weightman MJ, Sirichai P, Jones A. How do junior medical officers use online information resources? a survey. BMC Biomed Educ. 2016;16:120. DOI: http://dx.doi.org/10.1186/s12909-016-0645-x.
Haines LL, Light J, O’Malley D, Delwiche FA. Information-seeking behavior of basic science researchers: implications for library services. J Med Libr Assoc. 2010 Jan;98(1):73–81. DOI: http://dx.doi.org/10.3163/1536-5050.98.1.019.
Kendall SK. Basic biomedical scientists: the rediscovered library users. Against the Grain. 2014 Apr;26(2):34–6. DOI: http://dx.doi.org/10.7771/2380-176X.6697.
Pennington B, Chapman S, Fry A, Deschenes A, Green McDonald C. Strategies to improve the user experience. Serials Rev. 2016;42(1):47–58. DOI: http://dx.doi.org/10.1080/00987913.2016.1140614.

## MLA INSIGHT INITIATIVE TASK FORCE

The task force is the steering committee for the multi-year InSight Initiative. The task force also reviews the applications from librarians expressing an interest to attend an InSight Summit and selects the participants based on the summit theme and a representative mix of librarians affiliated with the diverse organizations with whom vendors work, including academic medical centers, community hospitals, specialty schools (nursing, pharmacy, etc.), governmental agencies, corporations, and nonprofit advocacy and community-based organizations.

Gerald J. Perry, AHIP, FMLA, University of Arizona, Chair, MLA Past President
Barbara A. Epstein, AHIP, FMLA, University of Pittsburgh, Member, MLA Past President
Michelle Kraft, AHIP, Cleveland Clinic Foundation, Member, MLA Past President
Gabriel R. Rios, Indiana University, Member
Daniel J. Doody, Doody Consulting, Summit Organizer
Rich Lampert, Doody Consulting, Summit Co-Organizer
Beverly Murphy, AHIP, FMLA, Duke University Medical Center, Board Liaison, MLA President
Kevin Baliozian, Medical Library Association, Member, MLA Executive Director
Mary M. Langman, Medical Library Association, Staff Liaison

## INSIGHT SUMMIT 2 PROGRAM COMMITTEE

The program committee developed the schedule and all program elements for InSight Summit 2. It was appointed by the InSight Initiative Task Force and consisted of three librarians, three representatives from participating organizations, the program facilitators, and a liaison from the InSight Initiative Task Force.

Daniel J. Doody, Doody Consulting, Summit Facilitator
Rich Lampert, Doody Consulting, Summit Facilitator
Emma Cryer Heet, Duke University, Member, Library Representative
Nadine Dexter, AHIP, University of Central Florida, Member, Library Representative, AAHSL Board Representative
Deborah Harris, F1000, Member, Industry Representative
Andrea Lopez, Annual Reviews, Member, Industry Representative
Elizabeth R. Lorbeer, AHIP, Western Michigan University, Member, Library Representative
Rob McKinney, *New England Journal of Medicine,* Member, Industry Representative
Michelle Kraft, AHIP, Cleveland Clinic Foundation, Liaison, InSight Initiative Task Force
Mary M. Langman, Medical Library Association, Staff Liaison

## INSIGHT SUMMIT 2 FACILITATORS

Discussions and group exercises were facilitated by InSight Summit 2 Program Committee members.

Daniel J. Doody, Doody Consulting
Rich Lampert, Doody Consulting
Michelle Kraft, AHIP, Cleveland Clinic Foundation

## INSIGHT SUMMIT 2 PARTICIPATING ORGANIZATIONS AND SPONSORS

MLA thanks the following participating organizations.

Annual Reviews
American Psychiatric Association Publishing
American Psychological Association
BMJ Publishing
Elsevier
F1000
The JAMA Network
McGraw-Hill Education
NEJM Group
Oxford University Press
ProQuest
Rockefeller University Press
Springer Nature
Wolters Kluwer

MLA also thanks the Association of Academic Health Sciences Libraries (AAHSL) and Elsevier for their financial support of the travel expenses and registration of librarian participants.

## INSIGHT SUMMIT 2 PARTICIPANTS

The InSight Summit had an equal representation of librarian leaders and participating organizations.

Katherine G. Akers, Wayne State University
Priya Arora, Wolters Kluwer
Nicole Capdarest-Arest, AHIP, University of California, Davis
Mark Chodash, Wolters Kluwer
Emma Cryer Heet, AHIP, Duke University
Vida Damijonaitis, JAMA Network
LaVentra E. Danquah, Wayne State University
Nadine Dexter, AHIP, University of Central Florida
Suzanne Fricke, AHIP, Washington State University
John Gallagher, Yale University
Terri Gotschall, AHIP, University of Central Florida
Susan Haering, Massachusetts Medical Society/NEJM Group
Deborah Harris, F1000
Lilian Hoffecker, University of Colorado
Theresa Hunt, Elsevier
Marc Iacono, Springer/Nature
Dawn Keech, ProQuest
Elizabeth A. Ketterman, East Carolina University
Shandra Lee Knight, National Jewish Health
Michelle Kraft, AHIP, Cleveland Clinic
Andrea Lopez, Annual Reviews
Elizabeth R. Lorbeer, AHIP, Western Michigan University
Gregory Malar, Rockefeller University Press
Robert McKinney, Massachusetts Medical Society/NEJM Group
Eve Melton, AHIP, Kaiser Permanente
David Nygren, American Psychological Association
Kevin O’Brien, University of Illinois at Chicago
Rikke Sarah Ogawa, AHIP, University of California, Los Angeles
Daniel Pickhardt, JAMA Network
Scott Pidgeon, Oxford University Press
Barbara A. Platts, AHIP, Munson Healthcare
Nicole Ridgeway, American Psychological Association
Ryan Rodriguez, BMJ
Laura Schimming, Icahn School of Medicine at Mount Sinai
Rebecca Seger, Oxford University Press
Amanda Sprochi, AHIP, University of Missouri
Janet Szczesny, ProQuest
Claire J. Twose, Johns Hopkins University
Megan Vance, American Psychiatric Association
Randall Watts, AHIP, University of Tennessee
Michael Weitz, McGraw-Hill Education
Martin Wood, AHIP, Florida State University

## References

[1] Akers KG. Report from the Medical Library Association’s InSight Initiative Summit 1: engaging users in a disruptive era. J Med Libr Assoc. 2018 Oct;106(4):554–72. DOI: 10.5195/jmla.2018.561.

[2] Gardner T, Inger S. How readers discover content in scholarly publications [Internet]. Renew Publishing Consultants; Aug 2018 [cited 19 Jan 2019]. <http://renewpublishingconsultants.com/wp-content/uploads/2017/07/How-Readers-Discover-Content-in-Scholarly-Publications.pdf>.

[3] Wray CM, Auerbach AD, Arora VM. The adoption of an online journal club to improve research dissemination and social media engagement among hospitalists. J Hosp Med. 2018 Nov;13(11):764–9. DOI: 10.12788/jhm.2987.

[4] Brian R, Orlov N, Werner D, Martin SK, Arora VM, Alkureishi M. Evaluating the impact of clinical librarians on clinical questions during inpatient rounds. J Med Libr Assoc. 2018 Apr;106(2):175–83. DOI: 10.5195/jmla.2018.254.

[5] Funderburg L. Born too soon. Life. 2000;May.

[6] Offit PA. Bad advice: or why celebrities, politicians, and activists aren’t your best source of health information. Columbia University Press: New York, NY; 2018.

[7] Anderson K. Focusing on value—102 things journal publishers do (2018 update). Scholarly Kitchen [Internet]. 6 Feb 2018 [cited 19 Jan 2019]. <https://scholarlykitchen.sspnet.org/2018/02/06/focusing-value-102-things-journal-publishers-2018-update/>.

[8] Ketterman E, Pidgeon S. Guest post: MLA InSight—how to buy whisky. Scholarly Kitchen [Internet]. 9 Jan 2019 [cited 19 Jan 2019]. <https://scholarlykitchen.sspnet.org/2019/01/09/guest-post-mla-insight-how-to-buy-whisky/>.

